# Pregnancy outcomes in women with immunoglobulin A nephropathy: a nationwide population-based cohort study

**DOI:** 10.1007/s40620-021-00979-2

**Published:** 2021-03-08

**Authors:** Simon Jarrick, Sigrid Lundberg, Olof Stephansson, Adina Symreng, Matteo Bottai, Jonas Höijer, Jonas F. Ludvigsson

**Affiliations:** 1 Department of Pediatrics, Örebro University Hospital, 701 85 Örebro, Sweden; 2 Faculty of Health and Medicine, Örebro University, Örebro, Sweden; 3 Department of Nephrology, Danderyd Hospital, Stockholm, Sweden; 4 Department of Clinical Sciences, Karolinska Institutet, Danderyd Hospital, Stockholm, Sweden; 5 Clinical Epidemiology Division, Department of Medicine, Solna, Karolinska Institutet, Stockholm, Sweden; 6 Division of Obstetrics and Gynecology, Department of Women’s and Children’s Health, Karolinska Institutet, Stockholm, Sweden; 7 Department of Medical Epidemiology and Biostatistics, Karolinska Institutet, Stockholm, Sweden; 8 Division of Biostatisitcs, Institute of Environmental Medicine, Karolinska Institutet, Stockholm, Sweden; 9 Division of Epidemiology and Public Health, School of Medicine, University of Nottingham, Nottingham, UK; 10 Department of Medicine, Columbia University College of Physicians and Surgeons, New York, NY USA

**Keywords:** IgA nephropathy, Pregnancy, Epidemiology, Glomerulonephritis, Prognosis

## Abstract

**Background:**

Immunoglobulin A nephropathy (IgAN) incidence peaks in childbearing age. Data on pregnancy outcomes in women with IgAN are limited.

**Methods:**

We performed a register-based cohort study in a nationwide cohort of women with biopsy-verified IgAN in Sweden, comparing 327 pregnancies in 208 women with biopsy-verified IgAN and 1060 pregnancies in a matched reference population of 622 women without IgAN, with secondary comparisons with sisters to IgAN women. Adverse pregnancy outcomes, identified by way of the Swedish Medical Birth Register, were compared through multivariable logistic regression and presented as adjusted odds ratios (aORs). Main outcome was preterm birth (< 37 weeks). Secondary outcomes were preeclampsia, small for gestational age (SGA), low 5-min Apgar score (< 7), fetal or infant loss, cesarean section, and gestational diabetes.

**Results:**

We found that IgAN was associated with an increased risk of preterm birth (13.1% vs 5.6%; aOR = 2.69; 95% confidence interval [CI] = 1.52–4.77), preeclampsia (13.8% vs 4.2%; aOR = 4.29; 95%CI = 2.42–7.62), SGA birth (16.0% vs 11.1%; aOR = 1.84; 95%CI = 1.17–2.88), and cesarean section (23.9% vs 16.2%; aOR = 1.74, 95%CI = 1.14–2.65). Absolute risks were low for intrauterine (0.6%) or neonatal (0%) death and for low 5-min Apgar score (1.5%), and did not differ from the reference population. Sibling comparisons suggested increased risks of preterm birth, preeclampsia, and SGA in IgAN, but not of cesarean section.

**Conclusion:**

We conclude that although most women with IgAN will have a favorable pregnancy outcome, they are at higher risk of preterm birth, preeclampsia and SGA. Intensified supervision during pregnancy is warranted.

**Supplementary Information:**

The online version contains supplementary material available at 10.1007/s40620-021-00979-2.

## Introduction

Immunoglobulin A nephropathy (IgAN) is the most common primary glomerulonephritis [1, 2] and a leading cause of the global burden of chronic kidney disease (CKD) [2, 3]. Incidence rates peak in (reproductive ages in) the third and fourth decades of life. Given that there is widespread agreement that CKD increases both maternal and fetal pregnancy risks [4, 5], pregnancy could be a major concern in women with IgAN. Available studies of pregnant women with IgAN have mainly focused on the effects that pregnancy might have on maternal renal function. As such, one systematic review showed no increased risk for adverse renal outcomes in predominantly early IgAN [6]. However, there is limited knowledge of IgAN impact on pregnancy outcomes. As recently reviewed by Piccoli et al. [7] an increased risk of preeclampsia, preterm birth, and low birth weight in IgAN pregnancies has been suggested. However, previous studies either lack pregnant reference individuals without IgAN [6, 8–12] or studied IgAN as part of a mixed exposure group with several renal diagnoses where only a minority of women were suffering from IgAN [13–15].

Through a national, population-based cohort of more than 4,000 patients with biopsy-proven IgAN [16, 17], we examined pregnancy outcomes in a subset of women giving birth between 1992 and 2011 compared with pregnant women from a matched reference population. Additionally, in a sibling design, we compared pregnancies in women with IgAN and pregnancies in their sisters without biopsy-verified IgAN, to account for the influence of potential intrafamilial confounding. We hypothesized that women with IgAN are at increased risk of adverse pregnancy outcomes compared to pregnant, matched reference individuals and to their pregnant sisters.

## Materials and methods

### Registers

The Swedish personal identity number (PIN) is a unique code assigned to every Swedish resident at birth or immigration enabling large-scale data linkages of multiple registers [18]. In this study, we linked data on biopsy-verified IgAN from Swedish pathology departments with the Swedish Medical Birth Register (MBR), the National Patient Register (NPR) and the Total Population Register (TPR).

The MBR [19] contains data on > 98% of all births in Sweden. Information, collected prospectively, includes maternal demographic data, reproductive history, and complications from the first antenatal visit (generally within 12 weeks of gestation) through delivery and the postnatal period. The register started in 1973, but in this study, we included pregnancies from January 1, 1992 until 2011 when data on smoking and body mass index (BMI, kg/m^2^) were consistently reported.

The NPR includes hospital stays since 1964, with national coverage since 1987. Hospital outpatient visits have been included since 2001. The register contains data on admission and discharge, main and additional diagnoses, and surgical procedures. The register has been validated and has a high diagnostic accuracy of 85–95% for most diagnoses [20].

The TPR [21] contains demographic data on all Swedish residents. This register also links first-degree relatives to all individuals born in Sweden since 1932 who were still alive in 1961.

### Definition of IgAN and selection of patients and reference individuals

**Fig. 1:**
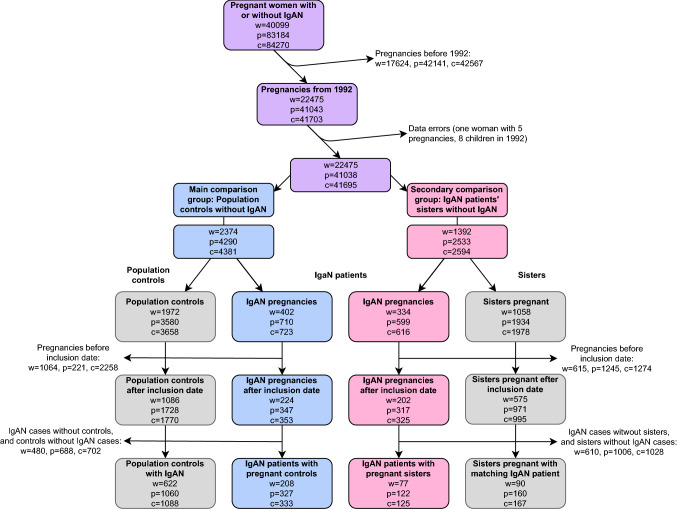
Flow chart showing patient inclusion and exclusion. *W*   women, *p *pregnancies, *c *children

We defined IgAN as having a biopsy record of IgAN (1974–2011) at any of the four pathology departments in Sweden where all renal biopsy specimens are evaluated (Stockholm, Göteborg, Linköping, and Malmö/Lund). Patients were ascertained by the local IT departments that searched biopsy records for the IgAN SnoMed CT (Systematized Nomenclature of Medicine—Clinical Term) codes D67300 (IgAN) and T71000 (kidney) [22]. Because one of the units (Malmö/Lund) did not provide SNOMED codes, biopsy reports from this region were reviewed manually (for a detailed description, see Welander et al. [17]). The resulting cohort of 4125 individuals with a biopsy report of IgAN was validated through manual review of complete patient charts from a random subset of 127 patients, confirming the diagnosis in 95% [16]. For each individual with IgAN, the government agency Statistics Sweden (SCB) identified up to five reference individuals from the TPR [21]. The reference individuals were matched to the IgAN patients for age, sex, calendar year, and county of residence to estimate relative risks of disease outcomes. None of the reference individuals had a diagnosis of IgAN at the time of inclusion. SCB also identified all siblings and spouses, as well as all first-degree relatives for both IgAN patients and reference individuals. The inclusion date was set as the date of the first renal biopsy in IgAN patients and the same date in siblings and matched reference individuals. This dataset was then linked to the MBR. All women with a registered pregnancy were eligible to participate. Pregnancies before January 1992 were excluded, as were all pregnancies before the inclusion date. Only pregnant IgAN women with at least one pregnant matched reference individual (primary analysis) or sister (secondary analysis) were included in the final analyses. Sisters with biopsy-verified IgAN were excluded (Fig. 1).

### Covariates

We obtained data on maternal year of delivery, maternal age, early pregnancy BMI, self-reported hypertension and smoking habits from the MBR. Women were asked about smoking at their first antenatal visit and classified into three categories of smoking status (0, 1–9, ≥ 10 cigarettes per day). For power reasons, however, the participants were eventually categorized into non-smokers and smokers (for definitions, see eTable S3). Data on educational level (as a proxy for socioeconomic status) were obtained from the TPR: ≤ 9 years (compulsory school), 10–12 years (upper secondary school), and ≥ 13 years (college or university). Data on pre-pregnancy diabetes mellitus and systemic inflammatory diseases were obtained from the MBR and the NPR (corresponding ICD codes are listed in eTable S1).

### Outcomes

Outcome data were obtained from the MBR. Main outcome was preterm birth before 37 full weeks of gestation. We also studied preeclampsia, small for gestational age, cesarean section, stillbirth or neonatal death (≤ 28 days postnatal age), low 5-min Apgar score (< 7) and gestational diabetes mellitus as secondary outcomes. Preterm birth was subdivided into spontaneous and medically indicated prematurity. Gestational diabetes mellitus and preeclampsia were defined as the occurrence of the corresponding ICD codes (supplementary table S2s) in the MBR or NPR from the start of pregnancy until 6 weeks after estimated delivery. We defined small for gestational age (SGA) as a birth weight below the 10th percentile using the ultrasound-based sex-specific Swedish reference curve for normal fetal growth (supplementary table S3). Data on end-stage renal disease (ESRD) at pregnancy start and during follow-up were obtained from the NPR (either chronic renal dialysis or kidney transplantation; see eTable S4 for corresponding ICD codes).

### Statistical analysis

Comparisons were made according to IgAN status, and matching was omitted to maximize statistical power (age, sex, and calendar period were instead controlled through statistical adjustment). All outcomes were analyzed as dichotomous variables and presented as absolute frequencies (*n*) and relative frequencies (%). To estimate whether IgAN was associated with an increased risk for adverse pregnancy outcomes, we used multivariable logistic regression with cluster-robust standard errors to calculate adjusted odds ratios (aORs). For fetal or infant outcomes (except stillbirth), only live deliveries were included and outcomes were analyzed per child. Maternal outcomes were analyzed per pregnancy. For gestational diabetes, we excluded women with pre-existing diabetes. The models were adjusted for calendar year of conception, maternal age, BMI, educational level, smoking status, pre-pregnancy diabetes type 1 or 2 (except in the analysis of gestational diabetes), other autoimmune diseases (eTable S1), and multiple gestation pregnancies. As a post-hoc sensitivity analysis, we also adjusted for pre-pregnancy hypertension (self-reported in the MBR), and performed a stratified analysis including only women without hypertension.

A post-hoc power analysis found that our study had an 80% power to detect a 1.44-fold increased risk of preterm birth at a significance level at 0.05.

We used Stata Statistical Software, Release 13 (College Station, TX: StataCorp, LP) for the statistical calculations. *P *values < 0.05 were considered statistically significant.

### Ethics

The study was approved by the Stockholm Ethics Review Board (January 22, 2014; Dnr 2013/2095-31/2). Because this was a strictly register-based study, informed consent was waived by the Board [23].

## Results

### Background

The primary analysis compared 208 women with IgAN and 622 reference individuals. The women with IgAN gave birth to 333 children after 327 pregnancies while the reference women without IgAN gave birth to 1088 children after 1060 pregnancies. In the secondary analysis, 77 women with IgAN and 125 children from 122 pregnancies were compared with their 90 sisters giving birth to 167 children from 160 pregnancies. Demographic data are presented in Table 1. Pre-gestational diabetes was more common in women with IgAN than in reference individuals (OR = 7.77; 95%CI = 2.00–30.2), but not compared with their sisters (OR = 1.07, 95%CI = 0.28–4.07). The distribution of age at conception, educational level, BMI, smoking status, parity and any systemic inflammatory disease other than IgAN was similar in women with IgAN and those without.

**Table 1:** Descriptive baseline characteristics of women with IgAN at first antenatal visit in Sweden in 1992–2015 and general population reference individuals, and women with IgAN and their sisters

	Pregnancies in women with IgAN (*n* = 333)	Pregnancies in population reference individuals (*n* = 1088)	Pregnancies in women with IgAN (*n* = 125)	Pregnancies in sisters (*n* = 167)
Year of conception				
Median (IQR)	2007 (2001, 2011)	2007 (2002, 2011)	2009 (2000, 2012)	2008 (2002, 2011)
Calendar period of conception				
1992–1999	64 (19.2%)	183 (16.8%)	30 (24%)	28 (16.8%)
2000–2007	110 (33%)	379 (34.8%)	28 (22.4%)	54 (32.3%)
2008–2015	159 (47.7%)	526 (48.3%)	67 (53.6%)	85 (50.9%)
Age at conception				
Median (IQR)	30 (27, 33)	31 (28, 34)	30 (28, 33)	30 (26, 34)
Mean (SD)	30.1 (4.65)	30.7 (4.43)	30.1 (4.14)	29.8 (5.19)
Educational level				
Compulsory school (0–9 years)	27 (8.1%)	64 (5.9%)	10 (8%)	4 (2.4%)
Upper sec. school (10–12 years)	137 (41.1%)	425 (39.1%)	51 (40.8%)	93 (55.7%)
College or university (≥ 13 years)	167 (50.2%)	599 (55.1%)	64 (51.2%)	70 (41.9%)
Missing	2 (0.6%)	0 (0%)	0 (0%)	0 (0%)
Parity				
Primiparous	126 (37.8%)	445 (40.9%)	47 (37.6%)	68 (40.7%)
Multiparous	207 (62.2%)	643 (59.1%)	78 (62.4%)	99 (59.3%)
Body mass index (BMI, kg/m^2^)				
Median (IQR)	23.9 (22, 27.1)	23.7 (21.6, 26.8)	23.3 (21.9, 25.0)	23.6 (21.5, 26.9)
Mean (SD)	25.1 (4.55)	24.6 (4.28)	24.4 (4.31)	24.4 (3.74)
Smoking status				
Non-smoker	289 (86.8%)	958 (88.1%)	110 (88%)	150 (89.8%)
Smoker	28 (8.4%)	68 (6.3%)	12 (9.6%)	7 (4.2%)
Missing	16 (4.8%)	62 (5.7%)	3 (2.4%)	10 (6%)
Hypertension (pre-pregnancy)				
No	299 (91.4%)	1058 (99.8%)		
Yes	28 (8.6%)	2 (0.2%)		
Diabetes (pre-pregnancy)				
No	326 (97.9%)	1085 (99.7%)	121 (96.8%)	162 (97%)
Yes	7 (2.1%)	3 (0.3%)	4 (3.2%)	5 (3%)
Other systemic inflammatory diseases				
No	325 (97.6%)	1078 (99.1%)	122 (97.6%)	159 (95.2%)
Yes	8 (2.4%)	10 (0.9%)	3 (2.4%)	8 (4.8%)
End-stage renal disease (ESRD)*				
Before first pregnancy	10 (3.2%)	0 (0.0%)		
During follow-up	4 (1.3%)	0 (0.0%)		

Continuous parameters are presented as mean values with standard deviations. Categorical values are presented as numbers and percentages. For all definitions, see main text or appendix

* See table S4 for definition according to ICD codes

### Pregnancy-related outcomes

Pregnancy-related outcomes are summarized in Table 2. Women with IgAN were at increased risk of preterm birth (13.1%), compared with reference women (5.6%; aOR = 2.69, 95%CI = 1.52–4.77); this was only found for medically indicated preterm birth (aOR = 6.55, 95%CI = 3.02–14.2), whereas spontaneous prematurity was similar between groups (aOR 1.04, 95%CI = 0.46–2.34). Adjusting also for hypertension altered the aOR for preterm birth only marginally (2.42, 95%CI = 1.32–4.42), as did the exclusion of hypertensive women (aOR 2.45, 95%CI = 1.33–4.49). The rate of very preterm birth before 34 weeks GA was not significantly increased compared to population controls. Women with IgAN had more than a four-fold higher risk of preeclampsia (13.8%) compared with reference individuals (4.2%; aOR = 4.29; 95%CI = 2.42–7.62). Infants born to women with IgAN were more often SGA (16%) compared with reference infants (11.1%; aOR = 1.84; 95%CI = 1.17–2.88). The risk of cesarean section was higher in women with IgAN compared with reference individuals. Rates of stillbirth were low and did not differ between women with IgAN (0.6%) and reference individuals (0.5%). No infants born to women with IgAN died in the neonatal period, and only three (0.3%) among reference individuals and one (0.6%) among sisters did. Gestational diabetes (< 1%) and fetal distress (as measured by a low 5-min Apgar score, < 2%) were rare both in women with IgAN and reference individuals. Similarly, sibling comparisons, although not statistically significant, suggested increased risks of preeclampsia, SGA, and preterm delivery in pregnancies complicated by IgAN. No association was found between maternal IgAN and cesarean section.

**Table 2:**
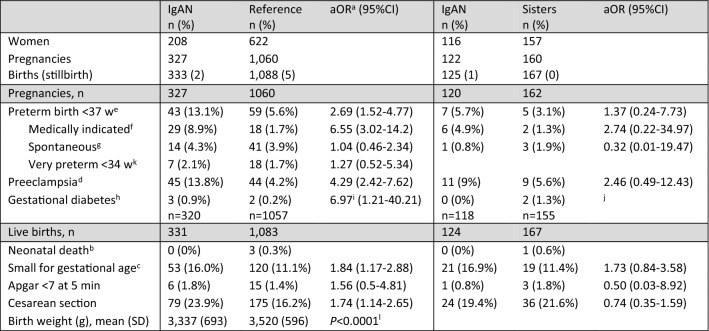
Maternal and infant outcomes in pregnant women with IgAN compared to general population controls and to their sisters

^a^Odds ratios adjusted for year of delivery, maternal age, body mass index (BMI), educational level, smoking (yes/no), pre-pregnancy diabetes (ICD8-9: 250, ICD10: E10-E11), other systemic inflammatory diseases (ICD codes in eTable S2), and multiple gestation pregnancies

^b^Infant death within 27 days postnatal age

^c^Birth weight below the 10th percentile for gestational week

^d^ICD9: 642E-H or ICD10: O14-O15 in the MBR or the NPR from pregnancy start to 6 weeks after estimated delivery

^e^Duration of pregnancy < 37 full weeks (≤ 258 days) in the MBR

^f^Code in the MBR for induction or cesarean section without the code for premature rupture of membranes

^g^All preterm deliveries not defined as induced

^h^ICD9: 648A or ICD10: O244 in the MBR or the NPR from pregnancy start to 6 weeks after estimated delivery. Women with pre-pregnancy diabetes mellitus (ICD8-9: 250 or ICD10: E10-E14) were excluded from the analysis

^i^Pre-pregnancy diabetes was not adjusted for because women with this diagnosis were excluded from these analyses

^j^Model not possible to estimate

^k^Duration of pregnancy < 34 full weeks (≤ 230 days) in the MBR

^l^Unpaired *t*-test

## Discussion

In this nationwide population-based cohort of more than 300 pregnancies in women with IgAN, we found an almost three-fold increased risk of preterm birth and a four-fold increased risk of preeclampsia. These findings were confirmed in sibling comparisons, although risk estimates were generally lower in these analyses, suggesting that genetic or environmental confounding factors shared within families might contribute to adverse pregnancy outcomes in women with IgAN.

### Comparison with previous studies

Our study is the largest study of pregnancy-related outcomes in women with IgAN and the only study using population-based reference individuals. Most studies have been restricted to tertiary centers that could introduce selection bias, which may explain reports of perinatal mortality as high as 2–3% even in high-income countries [10, 11]. In contrast, intrauterine death was seen in only 0.6% of the pregnancies in women with IgAN in our study, and none of the offspring of IgAN mothers died < 28 days postnatal age. These findings are both important and comforting to women with IgAN in fertile age.

### Strengths and limitations

The most obvious strength of our study is its large size (327 pregnancies in more than 200 women with IgAN). This resulted in a substantial statistical power to detect even small variations in risk. Moreover, we included a reference group of pregnant women who were selected from the same population as the IgAN women. This reference group allowed direct comparisons of pregnancy outcomes with adjustment for important confounders associated with adverse pregnancy outcomes (e.g., maternal age, smoking, BMI, and education). Because our study is population-based with national coverage, it should increase generalizability, at least within a high-income country context.

We used biopsy reports to ascertain IgAN. This approach has high sensitivity given that renal biopsy is mandatory for the diagnosis. Still, some patients, especially those with less severe disease, may go undetected. Biopsy reports for IgAN also have high specificity, as shown in our recent patient chart review. Of the 127 randomly selected patients, 121 had a clinical diagnosis of IgAN, corresponding to a positive predictive value of 95%. IgA deposits were reported in 97% of the biopsies and C3 deposits were seen in 89% of the patients [16]. Moreover, the MBR has a high coverage rate with only 0.5–5.0% missing records. For instance, more than 95% of pregnant Swedish women undergo early second trimester ultrasound to enable calculations of gestational age [19]. Finally, the TPR permitted the identification of pregnancies in sisters of women with IgAN. Pregnancy outcomes are influenced by a number of factors, some of which are subtle and not recorded in routine health care data or even possible to measure (e.g., health awareness and physical activity). Through our sibling comparisons, we could minimize the impact of intrafamilial confounding, and importantly, the positive association with adverse pregnancy outcome persisted for preeclampsia and SGA, albeit failing to reach statistical significance because of limited statistical power to detect population differences. The somewhat lower risk estimates in sibling comparisons may also signal the presence of residual confounding and shared risk factors, and that some part of the risk increase in IgAN women might be due to genetic or environmental risk factors shared with their sisters without IgAN.

Our study has some limitations. First, because our database does not include data on glomerular function or proteinuria, we could not stratify for IgAN disease activity or severity. Previous studies have suggested a correlation between adverse pregnancy outcomes (e.g., preeclampsia, preterm birth, low birth weight, and SGA) and impaired glomerular function [24]. Second, we were unable to stratify our patients according to underlying cause for consultation. In our patient chart review, macroscopic hematuria (29%) and urinary screening (26%) were the two most common reasons for IgAN work-up [16].

### Mechanisms

Glomerular diseases, including IgAN, are associated with high rates of hypertension and proteinuria. Not surprisingly, IgAN patients are at increased risk for vascular disease [25]. As such, IgAN during pregnancy shares characteristics with preeclampsia, which may be difficult to distinguish from features of the primary glomerular disease [26]. IgAN appears to bear a particularly high risk of preeclampsia [14], as witnessed by the more than four-fold increased risk in our cohort. Because preeclampsia is a main risk factor for adverse pregnancy outcomes, including preterm birth and intrauterine growth restriction, a similar pattern could be expected in IgAN. Previous studies have indeed indicated elevated risks of preterm birth and intrauterine growth restriction or SGA in CKD, increasing with CKD stage and with hypertension and high-grade proteinuria in CKD stage 1 [14, 15, 27]. Still, a sub-classification of preeclampsia has been suggested into an early (< 34 weeks of gestation) “placental” phenotype, depending on a primary abnormal placentation and the subsequent release into the bloodstream of vasoactive substances, and a late (> 34 weeks) “maternal” phenotype in which pre-existing endothelial dysfunction in women with hypertensive disease interacts with a primarily normal placenta. The placental phenotypes have been associated with much higher risks of adverse pregnancy outcomes, including a two-fold increased risk of SGA [28, 29]. The maternal phenotype could be expected to dominate in women with IgAN, who carry a high risk of hypertension, proteinuria, and vascular dysfunction before pregnancy [25]. This view is supported by a recent meta-analysis, comparing pregnancy in IgAN with pregnancy in a low-risk reference population, demonstrating a low SGA frequency (10.4%, non-significant OR = 1.27) in IgAN women despite a very high presence of preeclampsia (15.1%, OR = 11.80) [24]. In contrast, our IgAN cohort, with slightly less preeclampsia (13.8%), had a high and significantly increased occurrence of SGA (16.0%, aOR = 1.83). The observed increase of SGA is even more pronounced than what could be expected solely from preeclampsia, indicating that there may be other pathways linking IgAN and SGA. A diagnosis of early “placental” preeclampsia also may be missed to some extent in IgAN due to already present proteinuria and hypertension, which are expected to increase after cessation of blockers of the renin angiotensin system due to the pregnancy.

We found a pronounced increase in medically indicated preterm births, whereas the rate of spontaneous preterm birth was similar between groups. Although we did not examine each case separately, this increase could probably be explained by high rates of preeclampsia and SGA among women with IgAN, as these are common causes for medically indicated labor before term gestation.

## Conclusion

Our findings suggest that although most women with IgAN will have a favorable pregnancy outcome, IgAN in pregnancy may be linked to preeclampsia, preterm birth, and SGA. Our data support extra counseling and supervision of pregnant women with IgAN.

## Supplementary Information

Below is the link to the electronic supplementary material.

Supplementary file1 (DOC 118 KB)

## Data Availability

Other researchers can be granted individual access to our data through the Swedish National Board of Health and Welfare.

## Abbreviations

aOR: Adjusted odds ratio
CKD: Chronic kidney disease
IgAN: Immunoglobulin A nephropathy
BMI: Body mass index
MBR: The Swedish Medical Birth Register
NPR: The Swedish National Patient Register
SGA: Small for gestational age
TPR: The Swedish Total Population Register
