# Supplementary material for: Pregnancy outcomes in women with immunoglobulin A nephropathy: a nationwide population-based cohort study
Source: J Nephrol. 2021 Mar 8;34(5):1591–8. doi: 10.1007/s40620-021-00979-2 (PMC8494659; doi:10.1007/s40620-021-00979-2)
Supplement: Supplementary file 1 — Supplementary file1 (DOC 118 KB) [file 40620_2021_979_MOESM1_ESM.doc]

# **SUPPLEMENTARY ONLINE APPENDIX**

# Pregnancy outcomes in women with immunoglobulin A nephropathy: A nationwide population-based cohort study

Simon Jarrick, MD, Sigrid Lundberg, MD, PhD, Olof Stephansson, MD, PhD, Adina Symreng, MD, PhD, Matteo Bottai, PhD, Jonas Höijer, MSc, Jonas F. Ludvigsson, MD, PhD

Table of Contents

| Page 1 | **eTable S1.** International classification of disease (ICD) codes for comorbidity before conception |
| --- | --- |
| Page 2 | **eTable S2.** ICD codes for preeclampsia and gestational diabetes mellitus. |
| Page 3 | **eTabel S3.** Definition of pregnancy outcomes |
| Page 4 | **eTable S4.** International classification of disease (ICD) codes for renal endpoints using hospital-based in- or outpatient diagnosis. |
| Page 5 | **STROBE statement checklist** |

**eTable S1.** Covariates. International classification of disease (ICD) codes for diabetes and other systemic inflammatory diseases.

| **Comorbidity before conception** | **ICD-8** | **ICD-9** | **ICD-10** |
| --- | --- | --- | --- |
| Diabetes mellitus | 250 | 250 | E10-14 |
| Other systemic inflammatory diseases (any of those below) |  |  |  |
| *-Psoriasis* | 696 (not 969,30) | 696 (not 696D) | L40 |
| *-SLE* | 734,1 | 710A | M32.1; M32.8-9 |
| *-Rheumatoid arthritis* | 712,3; 714,93 | 714 | M05-M06; M08-M09; M12.3 |
| *-Crohn’s disease* | 563,00 | 555 | K50 |
| *-Ulcerative colitis* | 563,10 | 556 | K51 |
| *-Thyroiditis* | 245 | 245A-X | E06 |
| *-Hyperthyroidism* | 242 | 242 | E05 |
| *-Sarcoidosis* | 135 | 135 | D86 |
| *-Primary biliary cirrhosis* |  | 571G | K74.3 |
| *-ANCA vasculitis and other vasculitis* | 446 | 446 | M31 |
| *-Celiac disease* | 269,0 | 579A | K90.0 |
| *-Pelvospondylitis* |  |  | M45.9 |
| *-Autoimmune hepatitis* | 573,08; 573,09 | 573D | K75.4-5; K75.9 |
| *-Primary sclerosing cholangitis* |  |  | K83.0A |

**eTable S2.** The pregnancy outcomes preeclampsia and diabetes mellitus were defined as the corresponding International classification of disease (ICD) codes in the MBR or the NPR from the date of the first antenatal visit until six weeks after estimated delivery.

|  | **ICD-9** | **ICD-10** | **MBR category** |
| --- | --- | --- | --- |
| Preeclampsia (MBR or NPR) | 642E-H | E10-14 | - |
| Gestational diabetes mellitus (GDM)* (MBR or NPR) | 648A | O24.4 | - |

**excluding before conception the occurrence in the NPR of the ICD codes 250 (ICD9) or E10-14 (ICD10). During the study period, GDM was generally defined in Sweden as a fasting whole blood glucose ≥6.1 mmol/l (plasma glucose ≥7.0 mmol/l), or a 2‐h blood glucose ≥9 mmol/l (plasma glucose ≥10.0 mmol/l) after a 75 g oral glucose tolerance test.*

**eTable S3.** Definition of pregnancy outcomes according to variables in the Medical Birth Register (MBR) or International Classification of Diseases (ICD) version 9 or 10 codes in the MBR or in the National Patient Register (NPR).

| **Outcomes** | **Definition** | **Data** |
| --- | --- | --- |
| **Fetal/infant outcome** | | |
| Stillbirth | DODFOD=1 or 2 | MBR |
| Neonatal death | DDAGAR ≤27 [and not DODFOD 1 or 2] | MBR |
| Apgar <7 at 5 minutes | APGAR5 <7 | MBR |
| Small for gestational age | Birth weight for gestational age <10^th^ percentile, according to the ultrasound-based sex-specific Swedish reference curve for normal fetal growth.^1^ Ultrasound measurement for fetal growth is performed in some 95% of pregnancies.^2^ When no ultrasound data were available, we calculated gestational age based on last menstrual period.  1. Marsal K, Persson PH, Larsen T*, et al.* Intrauterine growth curves based on ultrasonically estimated foetal weights. *Acta Paediatr* 1996; **85:** 843-848.  2. Hogberg U, Larsson N. Early dating by ultrasound and perinatal outcome. A cohort study. *Acta Obstet Gynecol Scand* 1997; **76:** 907-912. | MBR |
| **Maternal outcomes** | | |
| Cesarean section | SECMARK=1 | MBR |
| Preterm birth | Pregnancy duration ≤258 days | MBR |
| Gestational diabetes^1^ | ICD9: 648A; ICD10: O244, during pregnancy (from preg.start to 6 weeks after estimated delivery); women with diabetes before conception (ICD9: 250 or ICD10: E10-14 in the NPR or MBR were excluded. | MBR or NPR |
| Preeclampsia^2^ | ICD9: 642E 642F 642G 642H; ICD10: O14 O15 during pregnancy (from pregnancy start to 6 weeks after estimated delivery) | MBR or NPR |

*^1^ During the study period, gestational diabetes was generally defined in Sweden as a fasting whole blood glucose ≥6.1 mmol/l (plasma glucose ≥7.0 mmol/l), or a 2‐h blood glucose ≥9 mmol/l (plasma glucose ≥10.0 mmol/l) after a 75 g oral glucose tolerance test (Hildén K, Hanson U, Persson M, et al. Overweight and obesity: a remaining problem in women treated for severe gestational diabetes.* Diabet Med *2016;* ***33:*** *1045-1051).*

*^2^ During the study period, preeclampsia was generally defined in Sweden as at least two blood pressure measurements ≥ 140/90 mmHg, combined with proteinuria (> 0.3 g/day or ≥ 1 + on a urine dipstick). The definition in women with preexisting hypertension or proteinuria was not consistent.*

**eTable S4.** International classification of disease (ICD) codes for renal endpoints using hospital-based in- or outpatient diagnosis.

| **Diagnostic group** | **ICD-8** | **ICD-9** | **ICD-10** | **Procedure codes** |
| --- | --- | --- | --- | --- |
| Medical diagnosis of end-stage renal disease (ESRD)^a^ | - | 585 | N18.0; N18.5 |  |
| Renal dialysis^b^ | Y29,01 | V45B; V56 | Z49; Z99.2 | 9200; V9200; 9212; V9212; 9314; V9531; DR012; DR013; DR016; DR024; QF006 |
|  |  |  |  | 9211; V9211; 9213; V9213; V9532; DR015; DR023; DR055; DV056 |
|  |  |  |  | 9219; V9219; 9223; V9223; DR017; DR020; DR055; DR056 |
| Renal transplantation |  | V42A | Z94.0 | 6070; KAS10; KAS20 |

^a^ In the Patient Register or the Cause of Death Register (underlying or contributory cause).

^b^ Regarded as ESRD if *occurring ≥3 times in a patient with ≥4 months between the first and last dialysis*.

**STROBE Statement – checklist of items:**

|  | Item No | Recommendation |
| --- | --- | --- |
| **Title and abstract** | 1✔ | (*a*) Indicate the study’s design with a commonly used term in the title **[p. 1]** |
|  |  | (*b*) Provide in the abstract an informative and balanced summary of what was done and what was found **[p. 5]** |
| Introduction |  | |
| Background/rationale | 2✔ | Explain the scientific background and rationale for the investigation being reported **[p. 6]** |
| Objectives | 3✔ | State specific objectives, including any prespecified hypotheses **[p. 6]** |
| Methods |  | |
| Study design | 4✔ | Present key elements of study design early in the paper **[p. 6]** |
| Setting | 5✔ | Describe the setting, locations, and relevant dates, including periods of recruitment, exposure, follow-up, and data collection **[p. 6-7]** |
| Participants | 6✔ | (*a*) Give the eligibility criteria, and the sources and methods of selection of participants. Describe methods of follow-up **[p 7-8]** |
|  |  | (*b*) For matched studies, give matching criteria and number of exposed and unexposed **[p. 7-8]** |
| Variables | 7✔ | Clearly define all outcomes, exposures, predictors, potential confounders, and effect modifiers. Give diagnostic criteria, if applicable **[p. 9]** |
| Data sources/ measurement | ✔8* | For each variable of interest, give sources of data and details of methods of assessment (measurement). Describe comparability of assessment methods if there is more than one group **[p. 9 + Supplementary tables]** |
| Bias | 9✔ | Describe any efforts to address potential sources of bias **[p. 10]** |
| Study size | 10✔ | Explain how the study size was arrived at  ***Comment***: This is a nationwide observational study, including all available patients with a biopsy report of IgA nephropathy and a pregnancy registered 1992-2011. We did not perform any *a priori* power analysis; a post-hoc power analysis for the main outcome is included in the manuscript **[p. 10]** |
| Quantitative variables | 11✔ | Explain how quantitative variables were handled in the analyses. If applicable, describe which groupings were chosen and why **[p. 10]** |
| Statistical methods | 12✔ | (*a*) Describe all statistical methods, including those used to control for confounding **[p. 10]** |
|  |  | (*b*) Describe any methods used to examine subgroups and interactions **[p. 10]** |
|  |  | (*c*) Explain how missing data were addressed **n/a** |
|  |  | (*d*) If applicable, explain how loss to follow-up was addressed **n/a** |
|  |  | (*e*) Describe any sensitivity analyses **[p. 10]** |
| Results |  | |
| Participants | ✔13* | (a) Report numbers of individuals at each stage of study—e.g., numbers potentially eligible, examined for eligibility, confirmed eligible, included in the study, completing follow-up, and analysed **[p. 11, fig. 1]** |
|  |  | (b) Give reasons for non-participation at each stage  ***Comment***: Because this was a strict registry-based study, study participants were not contacted (all data were analyzed without knowledge of the identity of the study participants). Hence, we had no “non-participation” at different stages of the study. |
|  |  | (c) Consider use of a flow diagram **[fig. 1]** |
| Descriptive data | ✔14* | (a) Give characteristics of study participants (e.g., demographic, clinical, social) and information on exposures and potential confounders **[p. 11 + table 1]** |
|  |  | (b) Indicate number of participants with missing data for each variable of interest **[table 1]** |
|  |  | (c) Summarize follow-up time (e.g. average and total amount) **[n. a.]** |
| Outcome data | ✔15* | Report numbers of outcome events or summary measures over time **[p. 11-12 + table 2]** |
| Main results | 16✔ | (*a*) Give unadjusted estimates and, if applicable, confounder-adjusted estimates and their precision (e.g., 95% confidence interval). Make clear which confounders were adjusted for and why they were included **[p. 11-12 + table 2]** |
|  |  | (*b*) Report category boundaries when continuous variables were categorized **table 1.** |
|  |  | (*c*) If relevant, consider translating estimates of relative risk into absolute risk for a meaningful period **n. a.** |
| Other analyses | 17✔ | Report other analyses done—e.g., analyses of subgroups and interactions, and sensitivity analyses **[p. 11-12 + table 2]** |
| Discussion |  | |
| Key results | 18✔ | Summarize key results with reference to study objectives **[p. 13]** |
| Limitations | 19✔ | Discuss limitations of the study considering sources of potential bias or imprecision. Discuss both direction and magnitude of any potential bias **[p. 13-15]** |
| Interpretation | 20✔ | Give a cautious overall interpretation of results considering objectives, limitations, multiplicity of analyses, results from similar studies, and other relevant evidence **[p. 13-15]** |
| Generalizability | 21✔ | Discuss the generalizability (external validity) of the study results **[p. 13-15]** |
| Other information |  | |
| Funding | 22✔ | Give the source of funding and the role of the funders for the present study and, if applicable, for the original study on which the present article is based **[p. 17]** |

*Give information separately for exposed and unexposed groups.

**Note:** An Explanation and Elaboration article discusses each checklist item and gives methodological background and published examples of transparent reporting. The STROBE checklist is best used in conjunction with this article (freely available on the Web sites of PLoS Medicine at http://www.plosmedicine.org/, Annals of Internal Medicine at http://www.annals.org/, and Epidemiology at http://www.epidem.com/). Information on the STROBE Initiative is available at http://www.strobe-statement.org.
